# Genomic Signatures of Experimental Adaptation to Antimicrobial Peptides in *Staphylococcus aureus*

**DOI:** 10.1534/g3.115.023622

**Published:** 2016-04-04

**Authors:** Paul R. Johnston, Adam J. Dobson, Jens Rolff

**Affiliations:** * Evolutionary Biology, Institute for Biology, Free University of Berlin, 14195 Berlin, Germany; † Berlin Center for Genomics in Biodiversity Research, 14195, Germany; ‡ Institute of Healthy Ageing, Genetics, Evolution and Environment, University College London, WC1E 6BT, UK; § Berlin-Brandenburg Institute of Advanced Biodiversity Research, 14195, Germany

**Keywords:** *Staphylococcus aureus*, antimicrobial peptide resistance, experimental evolution, Genetics of Immunity

## Abstract

The evolution of resistance against antimicrobial peptides has long been considered unlikely due to their mechanism of action, yet experimental selection with antimicrobial peptides (AMPs) results in rapid evolution of resistance in several species of bacteria. Although numerous studies have utilized mutant screens to identify loci that determine AMP susceptibility, there is a dearth of data concerning the genomic changes that accompany experimental evolution of AMP resistance. Using genome resequencing, we analyzed the mutations that arose during experimental evolution of resistance to the cationic AMPs iseganan, melittin, and pexiganan, as well as to a combination of melittin and pexiganan, or to the aminoglycoside antibiotic streptomycin. Analysis of 17 independently replicated *Staphylococcus aureus* selection lines, including unselected controls, showed that each AMP selected for mutations at distinct loci. We identify mutations in genes involved in the synthesis and maintenance of the cell envelope. These include genes previously identified from mutant screens for AMP resistance, and genes involved in the response to AMPs and cell-wall-active antibiotics. Furthermore, transposon insertion mutants were used to verify that a number of the identified genes are directly involved in determining AMP susceptibility. Strains selected for AMP resistance under controlled experimental evolution displayed consistent AMP-specific mutations in genes that determine AMP susceptibility. This suggests that different routes to evolve resistance are favored within a controlled genetic background.

Antimicrobial peptides (AMPs), ubiquitous in multicellular organisms (Zasloff 2002), are considered to be a promising source of new and potent antibiotics (Nguyen *et al.* 2011). Current research on AMPs focuses mostly on the mechanisms of action, and on the development of therapeutics, whereas only a small number of studies have addressed the important problem of bacterial resistance evolution. Resistance against cationic AMPs evolves readily *in vitro* in *Escherichia coli* and *Pseudomonas aeruginosa* (Perron *et al.* 2006), *Salmonella enterica* (Lofton *et al.* 2013), and *Staphylococcus aureus* (Habets and Brockhurst 2012; Dobson *et al.* 2013). Experimentally evolved strains of *S. aureus* that were selected successfully for resistance against the catioinc protegrin-1 analog iseganan (Dobson *et al.* 2013) survive better in a model host (Dobson *et al.* 2014) that relies heavily on AMPs to deal with long-lasting infections (Johnston *et al.* 2014). *S. aureus* populations selected for resistance to pexiganan and mellitin also show a trend toward increased survival in the host (Dobson *et al.* 2014). Here, we present a genomic analysis of *S. aureus* strains from these populations (Dobson *et al.* 2013), together with susceptibility data from transposon insertion mutants showing that a number of the identified genes are directly involved in mediating AMP susceptibility.

## Materials and Methods

Strains were isolated as single colonies from populations that were created by selecting *S. aureus* JLA513 (Shaw *et al.* 2006) (*hla-lacZ hla*+, derived from SH1000, from Simon Foster, University of Sheffield) for 28 d with increasing concentrations of AMPs, or with the aminoglycoside antibiotic streptomycin (Dobson *et al.* 2013). Streptomycin-selected strains are included here as a positive control since the genetic basis of streptomycin resistance is well-characterized in *S. aureus*. Briefly, to ensure adaptation to the culture medium, 50 µl of *S. aureus* JLA513 culture was passaged serially every 24 hr for 10 d in 5 ml Müller-Hinton Broth (MHB). Subsequently, five parallel selection lines were established in each treatment at minimum inhibitory concentration required to inhibit the growth of 50% of organisms (MIC_50_) (as well as unselected controls, which were serially passaged without exposure to AMPs or antibiotics) by inoculating 5 µl of serially passaged culture into 500 µl of MHB containing the cognate selective agent. A 5-µl aliquot of each 24-hr culture was passaged daily to fresh MHB. The concentrations of the selective agents were doubled each week for a total of 4 wk. See Supplemental Material, File S1 and Table S1 for full details and precise concentrations (Dobson *et al.* 2013). Strains were isolated from each of three independently selected replicate populations per selective agent (with the exception of iseganan-selected populations, where only two frozen population stocks remained viable), as well as from unselected controls, and the ancestral strain JLA513. Only strains derived from single colonies were sequenced; therefore, we cannot infer the frequency of a given mutation within the population of origin. MIC were calculated for the selective agents (Table S1) in 96-well plates as previously described (Andrews 2001), and DNA was isolated from each strain using a Roboklon DNA extraction kit (Roboklon GmbH, Germany). Genomic DNA from each strain was sequenced for 180 cycles using a HiSeq2000 by the Beijing Genomics Institute (BGI), resulting in 90-bp paired-end reads. Sequence data are available from the NCBI SRA under BioSample accession PRJNA291485. Strain JLA513 (Shaw *et al.* 2006) was constructed using strain SH1000, which is a derivative of strain 8325. The genetic differences between SH1000 and other members of the 8325 lineage have been described using both array-based resequencing (O’Neill 2010), and subsequently by *de novo* genome sequencing (Bæk *et al.* 2013). The differences comprise: the excision of three prophages from 8325 (Φ11, 12, 13), 13 single-nucleotide polymorphisms (SNPs; two synonymous, 11 nonsynonymous), a 63-bp deletion in the *spa-sarS* intergenic region, and an 11-bp deletion in *rsbU* (Bæk *et al.* 2013). Therefore, a consensus reference genome was first produced to account for these differences. Reads from JLA513 were assembled using SPAdes (Bankevich *et al.* 2012), and the resulting contigs were used to correct for the three phage excision sites in the 8325 reference genome. JLA513 reads were then mapped to the resulting sequence and bcftools consensus (H. Li *et al.* 2009) was used to correct the remaining 13 SNPs and two indels. To identify mutations in the selection lines, reads were mapped to this reference genome using BWA (Li and Durbin 2009) and sorted, deduplicated (to account for optical- and PCR-duplicates), and indexed using SAMtools (H. Li *et al.* 2009) and Picard (http://broadinstitute.github.io/picard). Average coverage was 134-fold (range 110- to 144-fold). Variants were called using FreeBayes version v0.9.14-8-g1618f7e (Garrison and Marth 2012), and coverage was calculated across 25-bp windows using IGVtools (Thorvaldsdóttir *et al.* 2013). All variants were independently verified using a second computational pipeline, breseq (Deatherage and Barrick 2014). Insertion mutants were obtained from the Nebraska Transposon Mutant Library (Fey *et al.* 2013) in order to test if the identified genes were directly involved in AMP resistance. MICs were calculated for each mutant, and the wild-type strain USA300_FPR3757 as described above.

### Data availability

The authors state that all data necessary for confirming the conclusions presented in the article are represented fully within the article.

## Results and Discussion

Between one and four mutations were identified per strain after accounting for differences between the JLA513 ancestor and the 8325 reference genome, and for mutations arising over the course of the experiment across treatments and unselected controls. In total, 28 mutations were identified across the 17 strains, including 24 nonsynonymous mutations affecting 13 genes, a segmental duplication of 124-kb region containing an entire *rrn* operon (Table 1 and Table S2) as well as one synonymous mutation, and two intergenic indels (Table S2).

**Table 1:** Mutations identified in strains selected for resistance to different antimicrobials

Selection	No. of Strains*^a^*	Gene	Function	Locus Tag*^b^*	Susceptibility of Tn Mutant*^c^*
IG	2	*yjbH*	Disulfide stress response	SAOUHSC_00938	Not tested
ML	1	*walR (yycG)*	Cell envelope biogenesis	SAOUHSC_00020	Not tested
ML	3	*rluD*-like	Pseudouridine synthase	SAOUHSC_00944	Unchanged
ML/PGML	3(2ML/1PGML)	*ytrA* ortholog	Cell wall stimulon	SAOUHSC_02155	Not tested
PG	1	*wcaG*	Nucleoside-diphosphate-sugar epimerase	SAOUHSC_00664	Unchanged
PG	2	*xdr*A	Xenobiotic response element	SAOUHSC_01979	Decreased
PG	2(1PG/1PGML)	*mgt (sgtB)*	Cell wall stimulon	SAOUHSC_02012	Decreased
PGML	1	*hpr*	Carbohydrate transport	SAOUHSC_01028	Not tested
PGML	1	*dak2*	Cell envelope biogenesis	SAOUHSC_01193	Increased
PGML	1	*putA (fadM)*	Amino acid metabolism	SAOUHSC_01884	Unchanged
STR	1	*nusA*	Transcription antitermination	SAOUHSC_01243	Not tested
STR	2	*glpK*	Glycerol kinase	SAOUHSC_01276	Unchanged
STR	1	*rrn* operons	Ribosome biogenesis	124-kb *rrn* region	Not tested
STR	3	*gidB (rsmG)*	Ribosome biogenesis	SAOUHSC_03051	Decreased

IG, iseganan; ML, melittin; PG, pexiganan; PGML, 1:1 wt/wt combination of melittin and pexiganan; STR, streptomycin. See Table S4 for further details on AMPs used. Three strains were sequenced for each of ML, PG, and PGML selections. Two strains were sequenced for IG.

a Number of strains with a mutation in a given gene.

b Identifier in *Staphylococcus aureus* NCTC 8325 reference genome.

c Susceptibility of transposon insertion mutants from the Nebraska Transposon Mutant Library to the cognate selective agent. Not tested, transposon mutant not available. See Table S3.

Pexiganan (PG) resistance was characterized by distinct nonsense mutations in the gene encoding the XRE-family transcriptional regulator XdrA in strains PG2.2 and PG4.2 (Table 1 and Table S2). XdrA was recently shown to activate transcription of *spa* (McCallum *et al.* 2010), which encodes the protein A virulence factor, and deletion mutants show increased β-lactam resistance (Ender *et al.* 2009). Here, a transposon mutant with an insertion in *xdrA* showed decreased pexiganan susceptibility (Table 1 and Table S3) indicating that XdrA is directly involved in pexiganan resistance. In addition to a mutation in *xdrA*, strain PG4.2 also carried a nonsynonymous substitution in *wcaG*, which encodes a putative UDP-glucose-4 epimerase (Table 1). Only a single mutation was observed in strain PG1.1, introducing a frameshift into *mgt* (*sgtB*), which encodes a monofunctional peptidoglycan glycosyltransferase (Table 1). A distinct nonsense in *mgt* was also identified in one pexiganan-melittin-selected (PGML) strain (see below) . An *mgt* transposon mutant was also found to be less susceptible to pexiganan (Table1 and Table S3). As part of the cell wall stimulon (Wang and Peery 2001), *mgt* is positively regulated by cell wall stress, and participates in the polymerization of lipid II into nascent peptidoglycan (Lovering *et al.* 2012). Recent work has shown that *mgt* mutations cause peptidoglycan chain length reduction as well as alterations in cellular morphology and division site placement (Rebets *et al.* 2014).

All three melittin- (ML) resistant strains were found to carry missense mutations resulting in either A35T or A35D substitutions in a gene encoding a putative RluD-like pseudouridylate synthase with no known role in antimicrobial susceptiblity. A transposon mutant from the Nebraska Transposon Mutant Library with an insertion in this gene showed no change in melittin susceptibility (Table 1 and Table S3). One melittin-resistant strain carried a L93I missense mutation in a region encoding an alpha helix immediately adjacent to the conserved active site quintet in the response regulator WalR (Table 1). WalKR regulates cell wall metabolism and is ubiquitous in the *Firmicutes*, where it is the only known essential two-component system (Dubrac *et al.* 2008). *walKR* mutations, including those affecting the WalR active site, arise during persistent clinical *S. aureus* infections and are known to confer resistance to vancomycin, and the lipopeptide antibiotic daptomycin by increasing the thickness of the cell wall (Howden *et al.* 2011). Identical nonsense mutations were identified in two melittin-resistant strains at the extreme 5′ end of the *ytrA* open reading frame, which encodes a winged helix-turn-helix GntR-family repressor (Table 1). Similar to its *B. subtilis* ortholog, *ytrA* is the first gene of an operon that encodes two putative ABC transporters. In *B. subtilis*, YtrA binds specifically to an inverted repeat in the *ytrA* and *ywoB* promoters, and transcription of the *ytr* and *ywo* operons is induced by cell-wall-active antibiotics, including the peptide antibiotics bacitracin, vancomycin, and ramnoplanin, with *ytrA* null mutations causing constitutive expression of both operons (Salzberg *et al.* 2011). Notably, the entire *ytrA* operon has been shown to be induced by cationic AMPs in *S. aureus*, where it is under negative regulation by the AMP sensing system *aps* (Li *et al.* 2007), and has also been implicated in nisin susceptibility in *S. aureus* SH1000 (Blake and O’Neill 2013). Although *ytrA* insertions are not present in the Nebraska Transposon Mutant Library, we were able to obtain two independent *ytr* operon transposon mutants with insertions downstream of *ytrA*, which did not show any detectable difference in AMP susceptiblity relative to the wild type (Table S3). This raises the possibility that the *ytrA*-null mutations observed here may mediate AMP susceptibility via derepression of the *S. aureus ywo* ortholog.

Iseganan (IG) resistance was associated with an identical 5-bp deletion in the extreme 3′ end of the *yjbH* gene in each of two strains from independent iseganan-selected lines (Table 1). YjbH controls the disulfide stress response by binding to the oxidative burst-specific transcriptional regulator Spx, and thereby controlling its degradation by the ClpXP protease (Göhring *et al.* 2011), a role that is conserved in *Bacillus subtilis* (Larsson *et al.* 2007). YjbH also modulates β-lactam susceptibility, with deletion mutants showing moderate resistance to various β-lactams. but not to the glycopeptide antibiotic vancomycin (Göhring *et al.* 2011). The precise mechanism by which YjbH modulates susceptibility is unknown. but is proposed to be a consequence of upregulation of PBP4. which results in increased peptidoglycan cross-linking (Göhring *et al.* 2011).

There were no common mutations identified in the genomes of three strains that were selected with a 1:1 wt/wt combination of pexiganan and melittin (Table 1). However, there were commonalities with strains that were selected with either melittin or pexiganan. A single missense mutation was identified in strain PGML3.2, which substitutes a conserved threonine residue in the winged helix-turn-helix DNA binding domain of YtrA (note that *ytrA* nonsense mutations were identified in two melittin-resistant strains described above). Similarly, a single nonsense mutation was identified in strain PGML5.1 in *mgt* (also mutated in 1 pexiganan-resistant strain described above). In contrast, three missense mutations were identified in the genome of a second pexiganan-melittin-selected strain. Interestingly, this included *dak2*, which encodes a dihydroxyacetone kinase responsible for incorporation of diphosphatidylglycerol into the cell membrane (M. Li *et al.* 2009). *dak2* was previously identified in a high throughput mutant screen for loci affecting susceptibility to the anionic human AMP dermcidin in *S. aureus* (M. Li *et al.* 2009). Mutations affecting the nonessential C-terminal DegV superfamily domain of Dak2 result in altered membrane phospholipid composition and decreased binding and activity of dermcidin but not of the catioinic human AMPs LL-37 or human β-defensin-3 (M. Li *et al.* 2009). Given this lack of cross-resistance to cationic AMPs in *dak2* mutants, Dak2-mediated susceptibility was thought to be specific to anionic AMPs such as dermcidin (M. Li *et al.* 2009). It is therefore surprising to find *dak2* mutation in response to selection with a combination of the cationic AMPs mellittin and pexiganan. Further evidence of the role of Dak2 in susceptibility to pexiganan and melittin was shown by increased susceptibility to both AMPs by a *dak2* transposon mutant (Table 1 and Table S3). Note that the *dak2* mutation reported here results in a G341D substitution, whereas the *dak2* mutant from the Nebraska Transposon Mutant Library is a transposon insertion mutant.

Mutations identified in streptomycin-selected strains occurred mostly in genes with known roles in streptomycin (STR) susceptibility (Table 1). Frameshift mutations in *gidB*, which encodes a 16S rRNA-specific 7-methylguanosine methyltransferase, were identified in all three streptomycin-selected strains (Table 1). In each case, the frameshift occurs within the region encoding the GidB methyltransferase domain. Mutations in *gidB* (*rsmG*) are associated with low-level streptomycin resistance in several species of bacteria including *S. aureus* (Mikheil *et al.* 2012; Verma *et al.* 2014; Wong *et al.* 2011; Okamoto *et al.* 2007), and it is speculated that loss of 16S methylation lowers the binding affinity of streptomycin, thus conferring the resistance phenotype (Wong *et al.* 2011). Here, a *gidB* transposon mutant was found to be fourfold less susceptible to streptomycin (Table 1 and Table S3). Two further mutations were identified that potentially affect ribosomal RNA. A 124-kb region containing an entire *rrn* operon appears to have been duplicated in a strain STR3.2, whereas strain STR1.1 carries a nonsynonymous substitution in the essential gene encoding NusA, which acts as an antiterminator for 16S rRNA transcription, as well as a chaperone for 16S rRNA folding (Bubunenko *et al.* 2013) (Table S2). Mutations were also identified in the glycerol kinase gene *glpK* in two strains (Table 1); however, a transposon insertion did not detectably alter streptomycin susceptibility (Table1 and Table S3).

Numerous studies have utilized mutant screens to identify loci that determine AMP susceptibility (Peschel *et al.* 1999; M. Li *et al.* 2009), but, with the exception of a single study (Lofton *et al.* 2013), there is a dearth of data concerning the genomic changes that accompany experimental evolution of AMP resistance, and whether the same loci are involved in each instance. Here, genome sequencing of strains isolated from independently replicated AMP selection lines identified mutations associated with AMP resistance evolution, and showed that each AMP selected for mutations at distinct loci. These mutations affected genes with known roles in susceptibility to AMPs and/or cell-wall-active antibiotics, as well as cell wall stress stimulon genes. All cationic AMPs used here form toroidal pores, yet there was little evidence of cross resistance or for mutations that were common across all AMP-selected strains, or even a single AMP, indicating that there are multiple routes to resistance. There is limited evidence of AMP-specific responses. For example, the staphylococcal virulence factor MprF determines susceptibility toward protegrins (*e.g.*, iseganan) but has little effect on magainin (pexiganan analog) or melittin susceptibility (Peschel *et al.* 2001). Also, little is known about AMP interactions with other constituents of the cell membrane, and whether these may contribute to the specificity observed here. A small number of mutations occurred in genes with no known role in antimicrobial susceptibility, such as the gene encoding the RluD-like pseudouridylate synthase, and may represent compensatory adaptations that warrant further study. Furthermore, mutations in the *walR* gene, such as that described here, are known to increase multidrug resistance, and to arise during clinical *S. aureus* infections (Howden *et al.* 2011). This is consistent with the notion that the evolution of resistance to AMPs may compromise host defenses against infection (Bell and Gouyon 2003; Habets and Brockhurst 2012; Makarova *et al.* 2016).

## 

## Supplementary Material

Supplemental Material
