# Supplementary material for: Genomic Signatures of Experimental Adaptation to Antimicrobial Peptides in Staphylococcus aureus
Source: G3 (Bethesda). 2016 Apr 4;6(6):1535–9. doi: 10.1534/g3.115.023622 (PMC4889650; doi:10.1534/g3.115.023622)
Supplement: Supplemental Material [file supp_6_6_1535__index.html]

Genomic Signatures of Experimental Adaptation to Antimicrobial Peptides in Staphylococcus aureus — Supplemental Material 

# Genomic Signatures of Experimental Adaptation to Antimicrobial Peptides in *Staphylococcus aureus*

## Supplemental Material for Johnston, Dobson, and Rolff, 2016

**Files in this Data Supplement:**

- Table S1 - MICs for various antimicrobials against 18 strains of *S. aureus*. (.pdf, 48 KB)
- Table S2 - Summary of all mutations. (.pdf, 63 KB)
- Table S3 - MICs for various antimicrobials against transposon insertion mutants of *S. aureus strain* USA300\_FPR3757 from the Nebraska Transposon Mutant Library.(.pdf, 72 KB)
- Table S4 - Details of antimicrobial peptides used. (.pdf, 72 KB)
- File S1 - Literate code used to perform analyses. (.pdf, 221 KB)
