# Supplementary material for: Genomic Signatures of Experimental Adaptation to Antimicrobial Peptides in Staphylococcus aureus
Source: G3 (Bethesda). 2016 Apr 4;6(6):1535–9. doi: 10.1534/g3.115.023622 (PMC4889650; doi:10.1534/g3.115.023622)
Supplement: Supplemental Material [file supp_g3.115.023622_TableS1.pdf]

TABLE S1. MICs for various antimicrobials against 18 strains of *S. aureus*.

	MIC(ug/ml)[a]				
Strain	Melittin	Pexiganan	Pex-Mel[b]	Streptomycin	Vancomycin
JLA513	8	8	8	4	2
IG1.2	4	8	8	4	2
IG2.1	4	8	8	4	2
ML1.1	32	8	32	8	4
ML4.2	32	8	16	4	2
ML5.2	32	16	16	4	2
PG1.1	8	16	8	4	2
PG2.2	4	16	8	4	2
PG4.2	4	16	8	8	2
PGML3.2	16	16	16	2	2
PGML4.4	8	32	16	4	2
PGML5.1	8	16	16	2	2
STR1.1	8	8	8	32	2
STR2.2	8	8	8	>64	2
STR3.2	8	16	8	>64	2
Uns1.1	4	4	4	4	2
Uns3.4	4	4	4	4	2
Uns4.2	8	8	8	8	2

[a]MIC, minimum antimicrobial concentration necessary to inhibit the growth of *S. aureus*.

[b]Equal quantities of pexiganan and melittin.

ML, melittin; PG, pexiganan; PGML, 1:1 wt/wt combination of melittin and pexiganan; STR, streptomycin; Uns, unselected control strain.
