# Supplementary material for: Genomic Signatures of Experimental Adaptation to Antimicrobial Peptides in Staphylococcus aureus
Source: G3 (Bethesda). 2016 Apr 4;6(6):1535–9. doi: 10.1534/g3.115.023622 (PMC4889650; doi:10.1534/g3.115.023622)
Supplement: Supplemental Material [file supp_g3.115.023622_TableS2.pdf]

TABLE S2. Summary of all mutations.

Strain	Mutation	Locus tag[a]	Annotation	Function
IG1.2	p.S266IfsX45	SAOUHSC_00938	*yjbH*	Disulfide stress response
IG2.1	p.S266IfsX45	SAOUHSC_00938	*yjbH*	Disulfide stress response
ML1.1	p.L93I	SAOUHSC_00020	*walR/yycG*	Cell envelope biogenesis; response regulator
ML1.1	p.A35T	SAOUHSC_00944	*rluD*-like	Pseudouridylate synthase
ML1.1	g.2101984_2101985insT	SAOUHSC_02270	intergenic	-
ML4.2	p.A35D	SAOUHSC_00944	*rluD*-like	Pseudouridylate synthase
ML4.2	p.L5X	SAOUHSC_02155	*ytrA*	Cell wall stimulon; repressor
ML5.2	p.A35D	SAOUHSC_00944	*rluD*-like	Pseudouridylate synthase
ML5.2	p.L5X	SAOUHSC_02155	*ytrA*	Cell wall stimulon; repressor
PG1.1	p.P39XfsX3	SAOUHSC_02012	*mgt/sgtB*	Cell wall stimulon; peptidoglycan glycosyltransferase
PG2.2	p.Q40RfsX24	SAOUHSC_01979	*xdr*A	Xenobiotic response element
PG4.2	p.M280V	SAOUHSC_00664	*wcaG*	Nucleoside-diphosphate-sugar epimerase; oxidoreductase
PG4.2	p.Q30X	SAOUHSC_01979	*xdr*A	Xenobiotic response element
PGML3.2	p.T74A	SAOUHSC_02155	*ytrA*	Cell wall stimulon; repressor
PGML4.4	p.A16D	SAOUHSC_01028	*hpr*	Carbohydrate transport
PGML4.4	p.G341D	SAOUHSC_01193	*dak2*	Cell envelope biogenesis; dihydroxyacetone kinase
PGML4.4	p.S138I	SAOUHSC_01884	*putA/fadM*	Amino acid metabolism; proline dehydrogenase
PGML5.1	p.Q251X	SAOUHSC_02012	*mgt/sgtB*	Cell wall stimulon; peptidoglycan glycosyltransferase
STR1.1	p.A227E	SAOUHSC_01243	*nusA*	Transcription antitermination; antiterminator
STR1.1	p.H87L	SAOUHSC_02727	NC_007795.1	Hypothetical protein; peptidase
STR1.1	p.R218DfsX75	SAOUHSC_03051	*gidB/rsmG*	Ribosome biogenesis; 16S rRNA methyltransferase
STR2.2	c.63A>G[b]	SAOUHSC_00489	*folP*	Dihydropteroate synthase
STR2.2	g.1090526_ 1090533del	intergenic	-	-
STR2.2	p.A332E	SAOUHSC_01276	*glpK*	Glycerolipid metabolism; glycerol kinase
STR2.2	p.S115EfsX12	SAOUHSC_03051	*gidB/rsmG*	Ribosome biogenesis; 16S rRNA methyltransferase
STR3.2	p.G251X	SAOUHSC_01276	*glpK*	Glycerolipid metabolism; glycerol kinase
STR3.2	g.2122437_2246248dup	segmental duplication	-	Encodes rRNA and ribosomal protein genes
STR3.2	p.S115EfsX12	SAOUHSC_03051	*gidB/rsmG*	Ribosome biogenesis; 16S rRNA methyltransferase

[a]Identifier in *Staphylococcus aureus* NCTC 8325 reference genome.

[b]Synonymous.

IG, iseganan; ML, melittin; PG, pexiganan; PGML, 1:1 wt/wt combination of melittin and pexiganan; STR, streptomycin.
