# Supplementary material for: Genomic Signatures of Experimental Adaptation to Antimicrobial Peptides in Staphylococcus aureus
Source: G3 (Bethesda). 2016 Apr 4;6(6):1535–9. doi: 10.1534/g3.115.023622 (PMC4889650; doi:10.1534/g3.115.023622)
Supplement: Supplemental Material [file supp_g3.115.023622_TableS3.pdf]

TABLE S3. MICs for various antimicrobials against transposon insertion mutants of *Staphylococcus aureus* strain USA300_FPR3757 from the Nebraska Transposon Mutant Library.

			MIC(ug/ml)[a]			
Strain	Locus tag[b]	Annotation	Melittin	Pexiganan	Pex-Mel[c]	Streptomycin
USA300	-	-	8	16	16	4
NE229	SAUSA300_1119	*dak2*	8	**8**	**8**	4
NE239	SAUSA300_1711	*putA* (*fadM*)	8	16	16	4
NE249	SAUSA300_2644	*gidB* (*rsmG*)	8	16	16	**16**
NE467	SAUSA300_0644	*wcaG*	8	16	16	4
NE596	SAUSA300_1855	*mgt* (*sgtB*)	8	**32**	16	4
NE822	SAUSA300_0909	*rluD*-like	8	16	16	4
NE896	SAUSA300_0903	*yjbH*	8	16	16	4
NE1023	SAUSA300_0984	*ptsI*	8	16	16	4
NE1445	SAUSA300_1797	*xdrA*	8	**32**	**8**	4
NE1587	SAUSA300_1192	*glpK*	8	16	16	4
NE1908[d]	SAUSA300_1911	ABC transporter	8	16	16	4
NE1188[d]	SAUSA300_1912	ABC transporter	8	16	16	4

[a]MIC(minimum inhibitory concentration), minimum antimicrobial concentration necessary to inhibit the growth of *S. aureus*.

[b]Identifier in *S. aureus* USA300_FPR3757 reference genome.

[c]Equal quantities of pexiganan and melittin.

[d]Insertions in the *ytr* operon downstream of *ytrA*. Insertions in *ytrA* are not present in the Nebraska Transposon Mutant Library.

ML, melittin; PG, pexiganan; PGML, 1:1 wt/wt combination of melittin and pexiganan; STR, streptomycin.
