# Supplementary material for: Genomic Signatures of Experimental Adaptation to Antimicrobial Peptides in Staphylococcus aureus
Source: G3 (Bethesda). 2016 Apr 4;6(6):1535–9. doi: 10.1534/g3.115.023622 (PMC4889650; doi:10.1534/g3.115.023622)
Supplement: Supplemental Material [file supp_g3.115.023622_TableS4.pdf]

TABLE S4. Details of antimicrobial peptides used.

AMP	Length (aa)	Net charge	Origin	Reference
Iseganan	17	+	Pig	Mosca et al. (2000)
Melittin	26	+	Honey bee	Raghuraman and Chattopadhyay (2007)
Pexiganan	22	+	Frog	Ge et al. (1999)

References

Mosca, D.; Hurst, M.; So, W. *Antimicrobial agents and chemotherapy* **2000**, *44*, 1803–1808.

Raghuraman, H.; Chattopadhyay, A. *Bioscience reports* **2007**, *27*, 189–223.

Ge, Y.; Macdonald, D. L.; Holroyd, K. J.; Thornsberry, C.; Wexler, H.; Zasloff, M.; Ge, Y.; Donald, D. L. M. A. C.; Holroyd, K. J.; Thornsberry, C.; Wexler, H.; Zasloff, M. *Antimicrobial agents and chemotherapy* **1999**, *43*, 782–788.
