# Supplementary material for: Genomic Signatures of Experimental Adaptation to Antimicrobial Peptides in Staphylococcus aureus
Source: G3 (Bethesda). 2016 Apr 4;6(6):1535–9. doi: 10.1534/g3.115.023622 (PMC4889650; doi:10.1534/g3.115.023622)
Supplement: Supplemental Material [file supp_g3.115.023622_FileS1.pdf]

FILE S1

Prepare reference genome

Download *Staphylococcus aureus* strain 8325 reference genome

Download reference from ensembl genomes 'ftp://ftp.ensemblgenomes.org/pub/bacteria/release-22/fasta/bacteria_18_collection/staphylococcus_aureus_subsp_aureus_nctc_8325/dna/index'.

```
mv Staphylococcus_aureus_subsp_aureus_nctc_8325.GCA_000013425.1.22.dna.fa 8325.fa
mv Staphylococcus_aureus_subsp_aureus_nctc_8325.GCA_000013425.1.22.gff3 8325.gff3
```

Account for differences between JL513 and 8325

Index 8325 reference genome and slice regions flanking prophages.

```
samtools faidx 8325.fa
samtools faidx 8325.fa Chromosome:1-1461779 > b1.fna
samtools faidx 8325.fa Chromosome:1509714-1922037 > b2.fna
samtools faidx 8325.fa Chromosome:1968147-2031604 > b3.fna
samtools faidx 8325.fa Chromosome:2075106-2821361 > b4.fna
```

Perform a `SPAdes` assembly with multiple values of k on JLA513 reads.

```
spades.py --pe1-1 jla513_1.fq.gz --pe1-2 jla513_2.fq.gz --careful -k 21,33,55,77,85,87,89 -t 12 -m 28 -o ./assembly && cd ./assembly
```

The assembly produced above can be downloaded here `http://dx.doi.org/10.6084/m9.figshare.1492404`. The following steps assume that this assembly is being used.

Extract contigs covering each excised prophage region and slice cognate regions. Contigs were identified by aligning the 8325 reference with the `SPAdes` assembly of strain JLA513 in `Mauve`.

```
pyfasta flatten assembly.fna
grep NODE_1_ assembly.fna -A1 > n1.fna
seqtk seq -r n1.fna > n1.rc.fna # correct the strand orientation of this contig.
samtools faidx n1.rc.fna
samtools faidx n1.rc.fna NODE_1_length_330729_cov_0.829198_ID_3:86459-88360 > ex1.fna
grep NODE_6_ assembly.fna -A1 > n6.fna
samtools faidx n6.fna
samtools faidx n6.fna NODE_6_length_112381_cov_1.46034_ID_19:59803-62296 > ex2.fna
grep NODE_16_ assembly.fna -A1 > n16.fna
samtools faidx n16.fna
samtools faidx n16.fna NODE_16_length_61500_cov_1.00241_ID_39:13002-13782 > ex3.fna
# concatenate fasta files in correct order and combine into a single entry.
cat b1.fna ex1.fna b2.fna ex2.fna b3.fna ex3.fna b4.fna > all.fna
echo '>minus_phage_1' > no_phi.fna
grep ">" -v all.fna | tr -dc '[:alpha:]' >> no_phi.fna
```

Mapping

Map the JLA513 reads to the modified reference and use the alignments to correct the remaining 13 SNPs and 2 indels.

```
bwa index -a bwtsw no_phi.fna
bwa mem -t 12 -M -R '@RG\tID:group1\tSM:jla513\tLB:jla513-lib1' no_phi.fna jla513_1.fq jla513_2.fq > jla513.sam
SortSam.jar INPUT=jla513.sam OUTPUT=jla513.bam SORT_ORDER=coordinate
MarkDuplicates.jar INPUT=jla513.bam OUTPUT=jla513.dedup.bam METRICS_FILE=jla513.metrics.txt
BuildBamIndex.jar INPUT=jla513.dedup.bam
freebayes --fasta-reference no_phi.fna jla513.dedup.bam --ploidy 1 --min-coverage 20 --use-mapping-quality --exclude-unobserved-genotypes --min-mapping-quality 1 --min-base-quality 3 --no-population-priors | vcffilter -f "QUAL > 20" > jla513.dedup.vcf
bcftools consensus -f no_phi.fna jla513.dedup.vcf.gz -o ref.fa
# annotate the reference genome
prokka --prefix ref ref.fa
bwa index -a bwtsw ref.fa
```

Mapping

Read group identifiers are required downstream, so they are included in the `bwa` call:

```
bwa mem -t 12 -M -R '@RG\tID:group1\tSM:IG-1-2\tLB:IG-1-2-lib1' ref.fa IG-1-2_FCC3NEJACXX_L6_WHAIPI002217-13_1.fq IG-1-2_FCC3NEJACXX_L6_WHAIPI002217-13_2.fq > IG-1-2.sam
bwa mem -t 12 -M -R '@RG\tID:group1\tSM:IG-2-1\tLB:IG-2-1-lib1' ref.fa IG-2-1_FCC3NEJACXX_L6_WHAIPI002218-14_1.fq IG-2-1_FCC3NEJACXX_L6_WHAIPI002218-14_2.fq > IG-2-1.sam
bwa mem -t 12 -M -R '@RG\tID:group1\tSM:ML-1-1\tLB:ML-1-1-lib1' ref.fa ML-1-1_FCC3NEJACXX_L6_WHAIPI002219-15_1.fq ML-1-1_FCC3NEJACXX_L6_WHAIPI002219-15_2.fq > ML-1-1.sam
bwa mem -t 12 -M -R '@RG\tID:group1\tSM:ML-4-2\tLB:ML-4-2-lib1' ref.fa ML-4-2_FCC3NEJACXX_L6_WHAIPI002220-16_1.fq ML-4-2_FCC3NEJACXX_L6_WHAIPI002220-16_2.fq > ML-4-2.sam
bwa mem -t 12 -M -R '@RG\tID:group1\tSM:ML-5-2\tLB:ML-5-2-lib1' ref.fa ML-5-2_FCC3NEJACXX_L6_WHAIPI002221-17_1.fq ML-5-2_FCC3NEJACXX_L6_WHAIPI002221-17_2.fq > ML-5-2.sam
bwa mem -t 12 -M -R '@RG\tID:group1\tSM:PG-1-1\tLB:PG-1-1-lib1' ref.fa PG-1-1_FCC3NEJACXX_L6_WHAIPI002222-18_1.fq PG-1-1_FCC3NEJACXX_L6_WHAIPI002222-18_2.fq > PG-1-1.sam
bwa mem -t 12 -M -R '@RG\tID:group1\tSM:PG-2-2\tLB:PG-2-2-lib1' ref.fa PG-2-2_FCC3NEJACXX_L6_WHAIPI002223-19_1.fq PG-2-2_FCC3NEJACXX_L6_WHAIPI002223-19_2.fq > PG-2-2.sam
bwa mem -t 12 -M -R '@RG\tID:group1\tSM:PG-4-2\tLB:PG-4-2-lib1' ref.fa PG-4-2_FCC3R7TACXX_L8_WHAIPI002592-20_1.fq PG-4-2_FCC3R7TACXX_L8_WHAIPI002592-20_2.fq > PG-4-2.sam
bwa mem -t 12 -M -R '@RG\tID:group1\tSM:PGML-3-2\tLB:PGML-3-2-lib1' ref.fa PGML-3-2_FCC3NEJACXX_L6_WHAIPI002224-21_1.fq PGML-3-2_FCC3NEJACXX_L6_WHAIPI002224-21_2.fq > PGML-3-2.sam
bwa mem -t 12 -M -R '@RG\tID:group1\tSM:PGML-4-4\tLB:PGML-4-4-lib1' ref.fa PGML-4-4_FCC3NEJACXX_L6_WHAIPI002225-22_1.fq PGML-4-4_FCC3NEJACXX_L6_WHAIPI002225-22_2.fq > PGML-4-4.sam
bwa mem -t 12 -M -R '@RG\tID:group1\tSM:PGML-5-1\tLB:PGML-5-1-lib1' ref.fa PGML-5-1_FCC3NEJACXX_L6_WHAIPI002226-23_1.fq
```

```
   PGML-5-1_FCC3NEJACXX_L6_WHAIPI002226-23_2.fq > PGML-5-1.sam
bwa mem -t 12 -M -R '@RG\tID:group1\tSM:St-1-1\tLB:St-1-1-lib1' ref.fa
   St-1-1_FCC3NEJACXX_L6_WHAIPI002230-27_1.fq
   St-1-1_FCC3NEJACXX_L6_WHAIPI002230-27_2.fq > St-1-1.sam
bwa mem -t 12 -M -R '@RG\tID:group1\tSM:St-2-2\tLB:St-2-2-lib1' ref.fa
   St-2-2_FCC3NEJACXX_L6_WHAIPI002231-29_1.fq
   St-2-2_FCC3NEJACXX_L6_WHAIPI002231-29_2.fq > St-2-2.sam
bwa mem -t 12 -M -R '@RG\tID:group1\tSM:St-3-2\tLB:St-3-2-lib1' ref.fa
   St-3-2_FCC3NEJACXX_L6_WHAIPI002232-30_1.fq
   St-3-2_FCC3NEJACXX_L6_WHAIPI002232-30_2.fq > St-3-2.sam
bwa mem -t 12 -M -R '@RG\tID:group1\tSM:Uns-1-1\tLB:Uns-1-1-lib1' ref.fa
   Uns-1-1_FCC3NEJACXX_L6_WHAIPI002233-31_1.fq
   Uns-1-1_FCC3NEJACXX_L6_WHAIPI002233-31_2.fq > Uns-1-1.sam
bwa mem -t 12 -M -R '@RG\tID:group1\tSM:Uns-3-4\tLB:Uns-3-4-lib1' ref.fa
   Uns-3-4_FCC3NEJACXX_L6_WHAIPI002234-32_1.fq
   Uns-3-4_FCC3NEJACXX_L6_WHAIPI002234-32_2.fq > Uns-3-4.sam
bwa mem -t 12 -M -R '@RG\tID:group1\tSM:Uns-4-2\tLB:Uns-4-2-lib1' ref.fa
   Uns-4-2_FCC3NEJACXX_L6_WHAIPI002235-33_1.fq
   Uns-4-2_FCC3NEJACXX_L6_WHAIPI002235-33_2.fq > Uns-4-2.sam
touch mapping.finished
```

Sort and de-duplicate

Sort and de-duplicate alignments:

```
for i in `cat sam.ids`
do
  SortSam.jar INPUT=$i.sam OUTPUT=$i.bam SORT_ORDER=coordinate
  MarkDuplicates.jar INPUT=$i.bam OUTPUT=$i.dedup.bam
     METRICS_FILE=$i.metrics.txt
  BuildBamIndex.jar INPUT=$i.dedup.bam
done
```

Variant calling

Call SNPs and indels separately.

```
for i in *.dedup.bam
do
  freebayes --fasta-reference ref.fa $i --ploidy 1 --no-indels
     --min-alternate-count 5 --min-coverage 30 --min-alternate-fraction 0.9
     --use-mapping-quality --exclude-unobserved-genotypes --min-mapping-quality
     20 --min-base-quality 20 --no-population-priors > $i.snps.vcf
  freebayes --fasta-reference ref.fa $i --ploidy 1 --no-snps
     --min-alternate-count 5 --min-coverage 30 --min-alternate-fraction 0.7
     --use-mapping-quality --exclude-unobserved-genotypes --min-mapping-quality
     20 --min-base-quality 20 --no-population-priors > $i.indels.vcf
done
```

Pull out SNPs that are not present in the unselected control strains:

```
mkdir -p coords/snps/ coords/indels/ cand/snps/ cand/indels/
# get all coordinates of snps or indels
grep Chromosome Uns*.dedup.bam.snps.vcf | cut -f2 | sort -ug >>
   coords/snps/uns.coords
grep Chromosome Uns*.dedup.bam.indels.vcf | cut -f2 | sort -ug >>
   coords/indels/uns.coords
```

```
# pull out coordinates that are not present in the unselected lines:
for i in `cat treatment_ids`
do
  grep -F -f coords/snps/uns.coords $i*.snps.vcf -w -v | grep Chromosome > cand/snps/$i*.cand
  grep -F -f coords/indels/uns.coords $i*.indels.vcf -w -v | grep Chromosome > cand/indels/$i*.cand
done
```

Manually verify the variants with samtools tview and IGV.

Verify the variants with `breseq` (depends on `bowtie2` and `R`).

```
for i in `cat list` # a list of read file prefixes (see Misc. below)
do
  # this uses the genbank file from the prokka annotation above.
  breseq -r ref.gbk ${i}_1.fq.gz ${i}_2.fq.gz -n $i -o $i
done
```

Coverage tracks

Average coverage over 25-bp windows using `igvtools`:

```
for i in `cat sam.ids`;do igvtools count $i.sorted.bam $i.sorted.bam.tdf; done
```

Misc.

```
$cat sam.ids

IG-1-2
IG-2-1
ML-1-1
ML-4-2
ML-5-2
PG-1-1
PG-2-2
PG-4-2
PGML-3-2
PGML-4-4
PGML-5-1
St-1-1
St-2-2
St-3-2
Uns-1-1
Uns-3-4
Uns-4-2
```

```
$cat list

IG-1-2_FCC3NEJACXX_L6
IG-2-1_FCC3NEJACXX_L6
ML-1-1_FCC3NEJACXX_L6
ML-4-2_FCC3NEJACXX_L6
ML-5-2_FCC3NEJACXX_L6
PG-1-1_FCC3NEJACXX_L6
PG-2-2_FCC3NEJACXX_L6
```

```
PG-4-2_FCC3R7TACXX_L8
PGML-3-2_FCC3NEJACXX_L6
PGML-4-4_FCC3NEJACXX_L6
PGML-5-1_FCC3NEJACXX_L6
St-1-1_FCC3NEJACXX_L6
St-2-2_FCC3NEJACXX_L6
St-3-2_FCC3NEJACXX_L6
Uns-1-1_FCC3NEJACXX_L6
Uns-3-4_FCC3NEJACXX_L6
Uns-4-2_FCC3NEJACXX_L6
```

References

1. Li, H., Handsaker, B., Wysoker,et al. (2009). The sequence alignment/map format and SAMtools. Bioinformatics, 25(16), 2078-2079.
2. Bankevich A, Nurk S, Antipov D, et al. SPAdes: A New Genome Assembly Algorithm and Its Applications to Single-Cell Sequencing. J Comput Biol 2012; 19: 455–77.
3. Li, H., & Durbin, R. (2009). Fast and accurate short read alignment with Burrows–Wheeler transform. Bioinformatics, 25(14), 1754-1760.
4. Seemann, T. (2014). Prokka: rapid prokaryotic genome annotation. Bioinformatics, btu153.
5. Garrison E, Marth G. Haplotype-based variant detection from short-read sequencing. arXiv preprint arXiv:1207.3907 [q-bio.GN] 2012
6. Deatherage DE, Barrick JE. Identification of mutations in laboratory-evolved microbes from next-generation sequencing data using breseq. Methods Mol Biol 2014; 1151: 165–88.
7. Langmead, B., & Salzberg, S. L. (2012). Fast gapped-read alignment with Bowtie 2. Nature methods, 9(4), 357-359.
